# Before the ban - an exploratory study of a local khat market in East London, U.K

**DOI:** 10.1186/s12954-015-0048-z

**Published:** 2015-06-12

**Authors:** Saba Kassim, Asha Dalsania, Johan Nordgren, Axel Klein, Josh Hulbert

**Affiliations:** Queen Mary, University of London, Barts and The London School of Medicine and Dentistry, Institute of Dentistry, 4 Newark Street, London, E1 2AT UK; Department of Social Work, Malmö University, Malmö, Sweden; Project Office with Egmont, Institute for International Relations, Brussels, Belgium; Drugscience, the Independent Scientific Committee on Drugs, London, UK

**Keywords:** Khat, Public health, Tower Hamlets, Drug policy, Khat market, Privileged access interviewer

## Abstract

**Background:**

Khat is a green leaf with amphetamine-like effects. It is primarily used among people in Africa, the Middle East and in the diaspora communities from these countries. Prior to the prohibition of khat in the UK on 24 June 2014, there was almost no information available on key aspects of the local khat market.

**Methods:**

A cross-sectional study was conducted in 2012 using snowball sampling, Privileged Access Interviewing and area mapping in order to identify khat sale establishments. Data was collected via face-to-face interviews using mixed methods for data collection. This included information about the establishments selling khat, khat pricing and its use among different ethnic minority groups, in addition to the potential sale of khat to children and risk assessment (e.g. use of pesticides on khat).

**Results:**

Five out of seven sellers identified agreed to participate. Sellers described their khat sale establishments as ‘community centres’ which included, for example, a restaurant basement. The sellers’ history of selling khat ranged between 1–15 years and khat’s sale took place between 2pm-10pm. *Miraa* (e.g. Lara) from Kenya was the most popularly used khat variety, sold in pre-wrapped bundles of approximately 250 g costing £3 each and delivered four days a week. *Harari* (e.g. Owdi) from Ethiopia was sold in 200 g, 400 g and 1 kg bundles, priced between £5 and £20 and delivered two days a week. The primary benefit of khat use was reported to be social interaction. The customers were predominantly adult males of Somali origin. Most sellers claimed a self-imposed ban on sales to children under 18 years old. Khat bundles had no labelling describing variety or weight and sellers had no knowledge of the use of pesticides on khat and did not advertise the risks associated with khat use.

**Conclusions:**

Khat selling establishments were businesses that did not adhere to trade standards regulations (e.g. labelling khat bundles). They claimed to provide a community service (facilitating social interaction) to their predominately Somali customers. Without a better understanding of the dynamics of the khat market there is a risk that both health and social needs of the vulnerable populations involved in the market continue to go unaddressed. Future research should track changes in the now illicit khat market in order to evaluate the social and public health implications following the recent changes to the current UK regulatory environment regarding khat.

**Electronic supplementary material:**

The online version of this article (doi:10.1186/s12954-015-0048-z) contains supplementary material, which is available to authorized users.

## Introduction

Khat leaves come from the evergreen shrub Catha edulis. In the UK, khat is chewed among diaspora communities from Eastern Africa and southern Arabia for pleasure and social interaction. The khat chew ritual facilitates community cohesion [1] and coping with adverse social conditions related to housing, migration and employment, war displacement and painful memories [2–6]. Other suggested benefits of khat use include as a pain and stress reliever [7, 8]. In the UK khat is used mainly by East African and Yemeni communities [1]. It has been argued that khat use poses risks to public health [9–12]. The khat use has been associated with several detrimental health effects, such as cardiovascular diseases and liver damage [3, 9, 13–15]. There are links with the use of other drugs (alcohol, cannabis with khat), but a strong coincidence with tobacco use [16–18]. Socio-economic impacts attributed to frequent khat use include family breakdown, and the financial burden of use [10, 11]. Recent reviews of the limited information-base have noted that as yet few of these harms are unequivocally established to be caused by khat use [3, 19].

The European Monitoring Centre for Drugs and Drug Addiction (EMCDDA) notes that most data collected about khat focuses on impacts of khat on health. However, the dynamics of the khat market itself (e.g. availability) and the environmental factors involved in mediating khat harms to users have not received adequate attention [20].

In the UK, khat is controlled as a Class C drug under the Misuse of Drugs Act 1971 [21], although the Advisory Council on the Misuse of Drugs (ACMD) found the available evidence on the social and physical harms of khat use limited, and advised against the criminalization of its users [22]. In prohibiting the khat, the UK government argued that “vulnerable communities” had to be protected, and noted that khat contains cathinone substances which are already controlled drugs, both in the UK and internationally [21]. Notably, the government stressed the need to align with “international partners” to prevent the UK from becoming a “single regional hub for the onward trafficking of khat” [23].

Prior to its prohibition in the UK, most khat was flown into Heathrow Airport from Kenya, Ethiopia and Yemen [24]. Khat was sold in bundles and varied in quantity per unit, quality and country of origin [22, 25, 26]. The most popular varieties were *Miraa* from Kenya and *Harari* from Ethiopia [3]. *Miraa* was preferred by Somali chewers [27], as it is rich in the principal stimulant alkaloid, cathinone [28]. In comparison, Yemenis preferred *Harari* khat [29] with less cathinone. Khat can be sourced as different varieties which are named after the place of cultivation and leaves’ potency [26, 30, 31] Fresh *Miraa* and *Harari* were transported to a warehouse in Southall after clearance from Heathrow Airport and wholesaled to sellers for approximately £35.00 a box. Dried leaves were sold at an average of £40.00 per box [3]. Forty bundles (5.5 kg) were usually found in each box of *Miraa* and 45 bundles (9 kg) in each box of *Harari* [24]. *Harari* and *Miraa* was sold for £5-6 and £3 respectively [24]. The imported value of the declared khat entering the UK (2.560 tons) for 2011–2012 was £13.8 million. The yield of Value Added Tax (VAT) was estimated to be £2.8 million [22, 24]. Local authorities seemed largely unaware (at least formally) of khat selling establishments, excepting Brent Council in London which declared khat cafes legitimate [32]. Licensing of khat establishments and weekly revenues of khat sellers were for the most part unrecorded. There was also a lack of knowledge of when khat arrived, where and how it was sold. Moreover, khat sales were advertised online [33] and the consumption of other commodities (e.g. soft drinks and tobacco) alongside khat has been reported elsewhere [1]. Little was known about khat sale advertisements and the sale of other commodities alongside khat, especially in the UK. Notably, there was a lack of information on the quality assurance of the khat supplied to the UK. Studies reported high levels of pesticides in blood samples of khat users and symptoms related to pesticide ingestion [34, 35]. This was corroborated by the fact that the khat grown in Ethiopia was reported to be treated with different and high levels of banned pesticides [36, 37]. Australia has imposed quality assurance for khat and importation is legal under licence [38].

Men were reported to be using khat [8, 10, 39] more commonly than women, possibly due to the social stigma attached to women chewing khat [27]. Information on the sale of khat to women was lacking. The ACMD [3] recommended that central government or local authorities explore the possibility of a voluntary agreement among khat sellers to prevent sales to minors. The London Borough of Brent barred the sale of khat to children under 16 years [32]. However, it remained unclear whether sellers in other areas were aware of this and had implemented similar restrictions.

Taking these aforementioned knowledge gaps into consideration, a survey of the khat market in the UK was important as it is impossible to develop robust policies concerning public health initiatives and harm reduction in the absence of evidence. In the context of the prohibition of khat in the UK, a lack of understanding of the availability and use of khat by immigrant communities could lead to social and health needs being neglected [20]. This study therefore aimed to establish the market for khat in East London, UK, with specific focus on the accessibility and availability of khat in Tower Hamlets. Objectives were as follows: 1) to identify areas of East London where khat is sold, 2) to identify the types of establishments selling khat, 3) to identify locations in which khat is sold and chewed, 4) to identify the form, brand and type of khat sold, 5) to identify the price variations of khat, 6) to identify when and how khat was delivered and sold, 7) to identify what other products were commonly sold with khat, and 8) to identify sales of khat within certain communities, and finally, to identify whether khat was adverted and being traded to children.

## Methods

### Study design

This study adopted an exploratory cross-sectional study design. It investigated a local khat market in the context of khat being traded and used as a legal commodity in the UK. Furthermore it offered an analysis of the legal khat market before its prohibition in 2014 [21]. The data was gathered in February and March 2012 from different khat selling establishments in East London, specifically in Tower Hamlets. This borough was chosen because previous studies reported significant numbers of people from khat chewing communities residing in this area [40, 41].

### Setting, recruitment and inclusion criteria of the study

Khat selling establishments, known as *mafrishes* amongst Somali and *Maqwati* amongst Yemeni, in Tower Hamlets were identified through privileged access interviewing, i.e. a community worker facilitated access [2, 42]. The area’s khat sellers were recruited through a snowball sampling and area mapping process [43, 44]. Arabic or English speaking khat sellers, aged 18 years and above, with permanent residency in the UK and free from health conditions that might have deterred their participation were included. Establishments were visited from 1:00 pm onward at times pre-specified by participating sellers. Data were collected through questionnaires administered face to face.

### Variables

The available literature was used by both S.Kassim and A. Dalsania to develop a questionnaire with six sections (Additional file 1) [45] as there was no available previous questionnaire on the khat market. Four sections of the questionnaire consisted of semi-structured questions that focused on describing the selling establishment, characteristics of khat customers (e.g. age and ethnicity), khat bundle [affordability and accessibility (e.g., origin and delivery days)] and risk assessment that included the use of pesticides in khat. The other two qualitative sections focused on sellers’ feedback, and participatory observations included visible adverts indicating the sale of khat, and whether children were buying khat.

The questionnaire was piloted and the study protocol was reviewed and approved by the Queen Mary University of London (QMUL) Research Ethics Committee (Ref: QREC2012/13). Participants were provided with an information sheet which outlined aspects of the study that included the aims and objectives of the study. Informed consent was then obtained prior to participation and participants were made aware that their participation was voluntary and that they could withdraw from the study at any time. Participants were given the option of not answering questions that they did not wish to. The confidentiality of the information obtained was emphasized, and complied with the Data Protection Act 1998. Each interview questionnaire was assigned a code number.

### Data analysis

The data was analysed using the Statistical Package for the Social Sciences (SPSS) version 20. Descriptive statistics were conducted to report information concerning aspects of the khat market. An opportunity was undertaken to calculate the estimated weekly and annually gross revenue and net profit made by each seller. Finally, a simple framework analysis [46] was used to analyse the questionnaire’s qualitative section. This analysis is used to synthesise the findings through sorting, categorisation, and interpretation of the collected qualitative data.

## Results

### Description of khat selling establishments and the selling of khat

From seven sellers identified, five male adult sellers agreed to participate; four with Somali backgrounds and one from Yemen. The establishments (Table 1) were in close proximity to each other and religious institutes (mosque) and amenities (shops, a market and restaurants) in Tower Hamlets in East London. Sellers described establishments as ‘community centres’ which varied in type from small living-room spaces with small seating areas to large shop's storage area (Table 1). Every establishment provided an area where male customers could socialise and consume the khat that they had purchased. The establishments relied upon word-of-mouth to promote their khat sales. The average length of time that each seller had been selling khat varied between 1 and 15 years, with the mean ± SD being 5.6 ± 5.98 years. All sellers were aware of other selling establishments and four sellers reported that though khat sales had remained static at their establishment aside from the short-term boost during the Muslim Eid, the number of khat selling establishments had increased.

**Table 1:** Calculated estimate gross and net profit from khat sale

Type of establishment	No^a^ of boxes of *Miraa* purchased	No^a^ of boxes of *Harari* purchased	Approx. bundle sales per day	Approx. Harari bundles sold per	Approx. *Miraa* bundles sold per day	Approx. bundle sales per week	Estimated gross revenue per week	Estimated net profit per week	Estimated gross revenue per year	Estimated net profit per year
Private home	0	7	45	45	0	280	£1,400	£403.20	£72,800	£20,966.40
Newsagent basement	10	0	50	0	50	400	£1,200	£500.00	£62,400	£26,000.00
Back of grocery shop	33	0	180	0	180	1320	£3,960	£1,650.00	£205,920	£85,800.00
Restaurant basement	12	5	100	20	80	680	£2,440	£925.00	£126,880	£48,100.00
Council flat	5	0	15	0	15	200	£600.00	£250.00	£31,200	£13,000.00
Total	60	12	390	65	325	2880	£9,600	£3728.20	£499,200	£193,866.40

^a^ Number of boxes purchased per week; each box of *Miraa* contained 45 bundles and *Harari* at 40 bundles

The number of khat bundles sold varied between sellers (Table 1). Only one khat seller sold both *Miraa* and *Harari* khat (Table 1). None of the establishments offered dried leaves or khat powder; although one seller reported that dried forms were available when air travel was compromised, i.e. during the Eyjafjallajökull volcano in April 2010. Table 1 reports estimated gross revenue and khat sellers self-report of net profit from khat sales. Finally, the daily demand and the weekly supply of khat matched up. The basis for this calculation is detailed in Appendix.

**Fig. 1:**
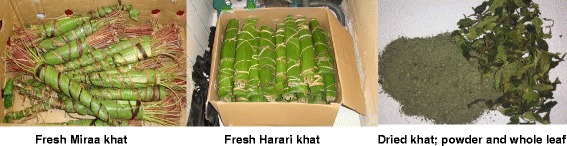
Types of khat sold in the United Kingdom. Fresh khat dominated before prohibition

As for the participatory observations, sellers did not advertise the sale of khat and there were no visible notices at the establishments to indicate khat sale. However, observing congregating men outside the khat selling establishments helped to identify the location. Sellers said they had a self-imposed age limit prohibiting children from buying khat. No children were seen buying khat whilst the researchers attended the establishments. We also did not see customers spit out khat on the pavement although there were cigarette butts, suggesting that the seller had requested that customers smoke outside and not in the establishment. *Harari* khat was displayed in fridges to keep the leaves fresh and *Miraa* was placed in cartons at the establishment’s selling point (Fig. 1).

### Characteristics of the customers

The most common ethnicity among customers for all of the establishments was Somali followed by Yemeni. Others included Ethiopians and some Caucasians. Sellers catered predominantly to adults between 18 and 65. All sellers reported that no children (under 18 years old) were allowed to purchase khat regardless of whether they were buying for older friends or relatives. Men comprised the bulk of the customers in all establishments. However, four sellers stated that they also had several female customers. On the pretext that there are religious proscriptions against the mixing of the sexes in public, these women were prohibited from sitting and chewing with men. Female customers did not enter the establishment and the transaction was done outside the establishment or via delivery.

### Availability, accessibility and affordability of khat

All sellers stated that the days when khat was most frequently bought were the days when khat was delivered to the establishments. *Miraa* sales were at their highest on Fridays, usually after prayers, followed by Sundays, Tuesdays and Wednesdays. *Harari* shipments arrived on Tuesdays and Fridays and it was on these days that its sales were highest. Most trade happened in the afternoons and evenings between 2 pm and 10 pm. None of the establishments used the internet to reserve and sell khat. Only one seller regularly took telephone reservations for khat before the arrival of a khat consignment.

The sales of *Aweday* and *Abo Mismar* (called *Mismary* amongst UK chewers) varieties of *Harari* khat from Ethiopia were reported. *Aweday* was the most popular *Harari* variety sold in 200 g, 400 g and 1 kg bundles with a price range from 5–20 pounds. *Miraa* came in different varieties (*Lara, Giza, Asli, Alele, Kangeta*), allegedly with different potencies and *Lara* was the most used in Tower Hamlets. *Miraa* was exclusively sold in pre-wrapped bundles of approximately 250 g for £3. Of the five khat sellers, three sold only *Miraa,* one *Harari* and one sold both *Harari* and *Miraa* khat (Table 1). Payment was made in cash, with four sellers offering a ‘pay later schemes’ or informal credit. This enabled customers to pay when they were able or to set up a monthly account. Water and soft drinks were frequently purchased alongside khat. Two sellers sold water only, another two also sold soft drinks. One seller did not sell anything other than khat.

### Risk assessment

The potentially harmful the use of pesticides on khat was unknown to most sellers. None of the establishments delivered any warnings on the packaging or at the point of sale on the possible risks associated with khat use. Three sellers, however, limited *Miraa* sales to 4–5 bundles per person. Two sellers stated that their customers could purchase unlimited amounts of khat.

### Perceived risks and benefits of khat use: Feedback from sellers

Sellers’ feedback about the benefits and risks of khat use was balanced. Sellers reported that social interaction was the main benefit besides relaxation, keeping the users updated about news from home and helping numerous customers, particularly those with limited English skills, with advice and help with paperwork. However, they reported that excessive khat use was associated with laziness, family stress, and financial burden as well as concurrent use of other drugs (e.g. alcohol). Sellers believed that the community should be educated about the potential harms of khat use and that a small number of problematic users gave the community a bad name.

## Discussion

This is the first study that has elucidated aspects of khat markets including availability, affordability and the promotion of ‘pay later schemes’ of khat in one specific area, namely the London Borough of Tower Hamlets, UK.

### What is known and what this study adds

The current literature reports that khat was accessed in places called *mafrishes.* These were café type establishments catering mainly to Somali communities [32]. They were either part of café or restaurant establishments or standalone places for buying and consuming khat. Notably they were considered amongst khat users as community meeting places [22, 27]. Our study has supported these findings and it showed that khat outlets can be concentrated in a local area; for example in our study, on one street in Tower Hamlets, close to local amenities.

The profile of khat availability to its local consumers was apparently not dissimilar from other drugs like smokeless tobacco, and its availability was found to be associated with the neighbourhood’s ethnic and socio-demographic composition [47]. The dominant sale of *Miraa* over other types of khat reflects the demographic composition of the neighbourhood; most customers were Somali men, with a few users from other minority backgrounds. Somali men chose *Miraa* over other types of khat because it was cheaper, with a reportedly stronger effect, making the users more talkative and ‘high’ [27, 28]. Women were reported to be buying their khat outside the establishments, reflecting the stigma surrounding female use. [27, 48]. Khat availability, as compared to reports in earlier studies [27, 32] had increased, apparently reflecting an increase in demand and changes in patterns of use [3]. Concerning the affordability of khat, a range of prices and bundle weights were reported by the sellers to match the needs of different users as reported elsewhere [3, 49, 50]. In accordance with the current literature, we recorded the facilitation of khat use among those without funds, via ‘pay later’ and credit [1, 27]. In addition, even though the sellers reported selling for a number of years, they did not observe any rise in their sales in terms of additional customers or higher volumes per customer, except for incidental surges, such as during Muslim Eid. This was the result of a rise in the number of competing outlets with khat available, as was documented elsewhere [27, 32]. This study was the first of its kind to quantify the profit that sellers claimed from khat sales.

With regard to the observational data, the cigarette butts discarded outside establishments constituted a “lack of health and safety measures’ and this finding was not dissimilar from other places [22, 27, 32]. The Conversely, they provide an indicator of rising awareness of the risks of indoor smoking and measures taken by mafrishes to reduce adverse health impact and inconvenience of second hand smoke. ACMD [3] has recommended against khat sales to children, and during our survey there was no evidence of minors trying to buy khat, or being prevented from doing so. The sellers reported a policy of not selling khat to anyone less than 18 years old, although we did not observe any formal signs stating this policy inside or outside outlets. One may consider the khat sellers’ approach as an informal self-regulation, amid considerations of their establishments as ‘community centres’. Word of mouth was reported as a way of locating khat establishments, however congregating of male khat users near establishments made it easy to spot khat establishments.

In terms of risk assessments and public health, although the literature reported that the sale of khat has been subjected to quality assurance in some countries [38] this was never imposed in the UK even though khat was a legal commodity. Out of five sellers, four reported not knowing where khat was obtained from and whether it was grown organically or treated with pesticides and fertilizers. However, khat sellers reported one of the risks of khat use was mixing it with other drugs (alcohol) which is in line with other studies [16]. Furthermore, no consumer guidance or warnings on the potential consequences of khat use was observed. Khat was sold in bundles wrapped in banana leaves with no information about the quality, content, variety and source, possible khat chewing risks or risk mitigation. Some sellers reported imposing limits on the number of khat bundles a customer could buy, but they did not advertise the risks associated with overconsumption. Sellers reported the sale of other commodities (water and soft drinks) alongside khat. Finally, feedback from sellers on the benefits and problems linked to khat chewing were in accordance with the existing literature [1, 19, 27].

### Strengths and limitations of the study, future research directions and policy implications

This study provided analyses of a local khat market before criminalisation, which can be compared with future studies about what is now an illicit khat market. Another strength of this study was the engagement of the khat sellers in providing this unique data which was previously reported as difficult to obtain [27]. However, this study had a number of limitations. The small sample size of participating sellers and a single site observation of a local khat market means that generalisation of the findings beyond this area is impossible. Significantly, a larger and representative sample could have provided a more detailed analysis of this local market. Therefore, the ‘saturation’ of the themes that emerged from the qualitative data of this study could be limited. Finally, the collection of self-reported data was not objectively verified (e.g. not selling to children) and the arrangement of the time for data collection to suit the preference of the khat sellers (pre-specified) meant that the findings need to be cautiously interpreted.

With a view to future research directions and policy implications, though it is now a criminal offence to import, buy, sell and possess khat [21] there is scant evidence that the criminalization of a substance and increased enforcement will reduce khat use [51–53]. It seems that the legal market for khat has turned to an illicit one requiring study. S.Kassim has observed the proliferation of sales of dried khat leaves and powder (Fig. 1) in London. This type of khat is rehydrated in tea or hot water, but the preservation of psychoactive properties are unclear, although at least some cathinone is stored in dry leaves [54]. This shift from fresh to dried khat has also been observed in Sweden, where the khat trade was criminalized in 1989, as the Swedish Customs intercepted 1.1 tons of dried khat during 2014 [55].

As noted before, several types of khat were sold in the studied market. Further research should be conducted about the transformation of khat from a legal commodity to an illicit one [56], as this might produce more costly, risky and unhealthy patterns of use and uncertainty regarding what kinds of khat are now dried and sold in powder form. The khat establishments have now lost their roles as ‘community centres’ and employment sources [57]. Therefore, future research should track alternative approaches that users and other khat market stakeholders may adopt [56–58]. Comparative analyses of local khat markets, worldwide, under different models of regulation may illuminate strategies to better manage the risks inherent in traditional and diversifying khat usage.

## Conclusions

We interviewed proprietors of khat selling establishments in Tower Hamlets, East London, and found that khat selling establishments were businesses that did not adhere to trade standards regulations (labelling khat bundles), and claimed to provide a community service (facilitating social interaction) to their predominantly Somali customers. Without a better understanding of the dynamics of the khat market there is a risk that both health and social needs of the vulnerable populations involved in the market continue to go unaddressed. Future research should track changes in the now illicit khat market, in order to evaluate the social and public health implications following the recent changes to the current UK regulatory environment regarding khat.

## Appendix

Calculation of weekly and annual revenues made by each seller; From HMRC figures and what was reported in this study, it is known that 40 bundles were typically found in each box of *Miraa* and 45 bundles in each box of *Harari*. Using this and the knowledge that *Harari* is sold for £5 and *Miraa* for £3, the estimate of weekly revenue for each establishment was calculated.

Khat sellers informally reported prices for both *Harari* and *Miraa* purchases and as such profits. *Harari*: it is purchased by weight at £15 per kg at wholesale price. A 45 bundle box with each typical bundle being 200 g would be 9 kg in weight. Therefore wholesale price of one box is £135. In addition another £10 is needed for customs and tax as well as an extra £15 per box for delivery. This equates to £160.00 per box. With 45 bundles of *Harari* selling for £5 each, the sellers make an estimated a profit of £65 per box. (£1.44 per bundle). *Miraa*: sellers state that with wholesale price, tax and delivery, one box of *Miraa* equates to £70. With 40 bundles of *Miraa* in each box selling for £3 a piece, sellers make approximately £50 per box. (£1.25 per bundle). Table 1 shows sellers’ self-reported net profit for khat sale.

## Additional file

Additional file 1:
**Interview Questionnaire - The Khat Market** [45].

## Abbreviations

PAI: Privileged Access Interviewer
HMRC: Her Majesty’s Revenue and Custom
ACMD: Advisory Council on the Misuse of Drugs
EMCDDA: The European Monitoring Centre for Drugs and Drug Addiction
