# Supplementary material for: Before the ban - an exploratory study of a local khat market in East London, U.K
Source: Harm Reduct J. 2015 Jun 12;12:19. doi: 10.1186/s12954-015-0048-z (PMC4465728; doi:10.1186/s12954-015-0048-z)
Supplement: Additional file 1: — Interview Questionnaire - The Khat Market [45]. [file 12954_2015_48_MOESM1_ESM.docx]

**Additional file 1**

**Saba Kassim^1*^**

*** Corresponding author**

**Phone: +44 (0) 207 8827374**

**Email: s.kassim@qmul.ac.uk,**

**Asha Dalsania^1^**

**Email:** [**asha.dalsania@southend.nhs.uk**](mailto:asha.dalsania@southend.nhs.uk)

**^1^ Queen Mary, University of London, Barts and The London School of Medicine and Dentistry, Institute of Dentistry, 4 Newark Street, London E1 2AT, UK**

**Interview Questionnaire - The Khat Market**

**Introduction:**

I am conducting a research study on how and where khat is sold in East London. To help me in this research, I will ask a few questions related to selling khat.

**Confidentiality:**

Your answers will be strictly confidential and will only be used to help me in my study. Your name will not be used on the form and you do not have to answer any questions that you do not wish to.

I am happy to answer any additional questions that you may have.

Thank you for helping me with my research.

**Interview Date:
Interview Time:**

**Section 1 - Establishment and khat selling**

1. Approximately how many years have you been selling khat?

______________________ Years

1. During this time have you increased the number of customers?

🞏 Yes

🞏 No

1. In your opinion do you think the sale of khat has increased in the area?

🞏 Yes

🞏 No

1. How would you describe your establishment?

🞏 Grocery Store/ Corner Shop

🞏 Cash and Carry

🞏 Market Stall

🞏 Restaurant

🞏 Coffee Shop

🞏 Community Centre

🞏 Private Home

🞏 Other (please specify) ______________________

1. Do you provide an area for customers to chew khat?

🞏 Yes

🞏 No

1. How do you advertise khat?

🞏 Word of mouth

🞏 Posters

🞏 Via other retailers

🞏 By Telephone

🞏 Online

🞏 Other (please specify) ______________________

1. Are you aware of where the khat comes from?

 Kenya (please specify area) ________________________

 Ethiopia (please specify area) ________________________

 Somalia (please specify area) ________________________

 Yemen (please specify area) ________________________

 Other (please specify) ________________________

1. On what days is khat delivered to your establishment?

| Day | Miraa | Harari | Other |
| --- | --- | --- | --- |
| Monday |  |  |  |
| Tuesday |  |  |  |
| Wednesday |  |  |  |
| Thursday |  |  |  |
| Friday |  |  |  |
| Saturday |  |  |  |
| Sunday |  |  |  |

1. Approximately how many bundles of khat do you purchase per week?

______________________

1. Approximately, how many khat bundles do you sell in a day?

______________________ Bundles

1. Does this change with the season/festivals (e.g. Eid)?

 Yes (please specify) ______________________

 No

1. What type of khat do you sell in your establishment?

 Miraa (please specify area of Kenya) ______________________

 Harari (please specify area of Ethiopia) ______________________

 Yemeni ((please specify area of Yemen) ______________________

 Other (please specify) ______________________

 Unsure

1. What Brands types of khat do you sell?

 Miraa ______________________

 Harari ______________________

 Yemeni ______________________

 Other (please specify) ______________________

1. What type of khat is the most popular among your customers?

______________________

1. In what form do you sell khat?

 Fresh leaves and Stems

 Dried leaves

 Powder

 Other (please specify) ______________________

1. How do you sell khat? By bundles, weight?

 Bundles

 Weight (kg)

 Other (please specify) ______________________

1. Do you offer different bundle sizes/different weight packages?

 Yes (please specify) ______________________

 No

1. Do you have a typical price per khat bundle/per given weight?

______________________

1. Do these prices vary by days of the week/different seasons? If so, by how much?

______________________

1. How do you store khat?

 In boxes

 In the refrigerator

 Other (please specify) ______________________

1. Do you know of any other places that sell khat in this area?

______________________

**Section 2 – About the customers**

1. Which ethnic background are most of your customers from?

 Somali

 Ethiopian

 Yemeni

 Kenyan

 Caucasian

 Other (please specify) ______________________

1. Approximately, what proportions of customers are from this background?

______________________ %

1. What age range do most of your customers fit into?

 Children

 Adults (18-65)

 Adults (65+)

1. Do children come to the establishment to collect khat on behalf of older friends/family members?

 No

 Yes

1. What gender are most of your customers?

 Male

 Female

1. How do most customers pay?

 Cash

 Credit Card/Debit Card

 Other (please specify) ______________________

1. Do you offer a ‘pay later’ service?

 No

 Yes

**Section 3 –** **Availability, accessibility and affordability of khat**

1. Between what hours is the most popular time to sell khat?

______________________

1. On what day would you say that you sell the most khat?

🞏 Monday

🞏 Tuesday

🞏 Wednesday

🞏 Thursday

🞏 Friday

🞏 Saturday

🞏 Sunday

1. Do you offer a delivery service?

🞏 No

🞏 Yes

1. Do seasons/festivals affect khat sales?

🞏 No

🞏 Yes (please specify) ______________________

1. Do you offer other means of selling/reserving khat?

🞏 By telephone

🞏 Online

🞏 Other (please specify) ______________________

1. Does your establishment have an age limit for selling khat?

🞏 Yes (please specify) ______________________

🞏 No

1. What is popularly purchased along with khat?

🞏 Snack Foods

🞏 Water

🞏 Milk

🞏 Soft Drinks (please specify) ________________________

🞏 Cigarettes

🞏 Other (please specify) ________________________

**Section 4- Risk assessment**

1. Do you have any warnings about khat in the selling establishment/on

the packaging?

 No

 Yes (please specify) ______________________

1. What is the maximum amount of khat you sell to a customer?

______________________

1. Are you aware of the quality of the khat you sell in your establishment?

 Yes, the khat is grown with fertilizers/pesticides

 Yes, the khat is grown organically

 I don’t know

**Section 5 – Feedback from khat sellers**

1. In your opinion, what are the main benefits of chewing khat?

__________________________________________________________________________________________________________________________________________________________________________________________

1. Do you think that khat chewing is a problem?

__________________________________________________________________________________________________________________________________________________________________________________________

1. Is there anything else you would like to add?

|  |
| --- |

**Section 6- Participatory observations**

1. Do customers spit out khat onto the pavement?

 No

 Yes

1. Are children buying khat?

 No

 Yes

1. Is there a visible advert to indicate that the establishment sells khat?

 No

 Yes
